# The Role of Phosphorus Fertilization in Antioxidant Responses of Drought-Stressed Common Beech and Sessile Oak Provenances

**DOI:** 10.3390/ijms26073053

**Published:** 2025-03-26

**Authors:** Antonia Vukmirović, Željko Škvorc, Saša Bogdan, Daniel Krstonošić, Ida Katičić Bogdan, Tomislav Karažija, Marko Bačurin, Magdalena Brener, Krunoslav Sever

**Affiliations:** 1 Faculty of Forestry and Wood Technology, University of Zagreb, Svetošimunska Cesta 23, HR-10000 Zagreb, Croatia; avukmiro@sumfak.unizg.hr (A.V.); zskvorc@sumfak.unizg.hr (Ž.Š.); sbogdan@sumfak.unizg.hr (S.B.); dkrstonosic@sumfak.unizg.hr (D.K.); ikaticic@sumfak.unizg.hr (I.K.B.); mbacurin@sumfak.unizg.hr (M.B.); mbrener@sumfak.unizg.hr (M.B.); 2 Faculty of Agriculture, University of Zagreb, Svetošimunska Cesta 25, HR-10000 Zagreb, Croatia; tkarazija@agr.hr

**Keywords:** *Fagus sylvatica*, *Quercus petraea*, drought, phosphorus fertilization, antioxidant enzyme activity, provenance-specific antioxidant response

## Abstract

During drought, a major abiotic stressor for European forests, excessive reactive oxygen species (ROS) are produced, causing oxidative damage that affects structural and metabolic tree functions. This research examines the effects of drought, phosphorus (P) fertilization, and provenance on photosynthetic pigments, malondialdehyde (MDA) concentrations, and antioxidant enzyme activities in common beech (*Fagus sylvatica* L.) and sessile oak (*Quercus petraea* (Matt.) Liebl.) saplings from two provenances. In a common garden experiment, four treatments were applied: regular watering with (+PW) and without P fertilization (−PW), and drought with (+PD) and without (−PD) P fertilization. Results showed that drought increased both MDA concentrations and antioxidant enzyme activity, particularly superoxide dismutase (SOD), catalase (CAT), peroxidase (POD), and ascorbate peroxidase (APX), which are responsible for ROS scavenging. Additionally, chlorophyll *a* + *b* concentrations were lower in drought-exposed plants. Phosphorus fertilization minimally affected MDA levels but enhanced antioxidant responses, particularly APX and CAT activities in oak during drought. Provenance differences were notable, with oak and beech from the drier provenance showing better adaptation, reflected in lower MDA levels and higher enzyme activities. This study underscores the importance of antioxidant defenses in coping with drought stress, with phosphorus fertilization and provenance shaping the species’ adaptive capacity.

## 1. Introduction

Drought, a stressful abiotic factor that negatively impacts various traits in forest trees, including physiological, morphological, and biochemical processes, has become a common subject of research in recent years [1,2,3,4,5,6,7,8]. During drought, photosynthesis is limited: one of the first defense mechanisms is stomata closure to prevent excessive water loss through transpiration. However, this also inhibits CO_2_ uptake, leading to an accumulation of excess energy as plants consume less light energy for photosynthetic carbon fixation [9]. This excess energy is redirected, leading to an increased flow of electrons to oxygen (O_2_), which results in cellular damage due to the overproduction of reactive oxygen species (ROS) [8]. These free radicals, such as hydrogen peroxide (H_2_O_2_), superoxide radical (O2^•^^−^), singlet oxygen (^1^O_2_), and hydroxyl radicals (OH^•^), are highly reactive and can damage cellular structures, impair normal functions, and disturb the redox balance [10]. They are highly damaging to all organelles, including chloroplasts, where photosynthesis takes place and one of the typical symptoms of oxidative stress is a reduction in photosynthetic pigment content [5,11]. Excessive ROS also damage the cell membrane, which is vital for sensing and responding to structural and metabolic disturbances [12]. The accumulation of ROS triggers lipid peroxidation, leading to oxidative damage to the membrane. The degree of lipid peroxidation is commonly assessed by measuring the concentration of malondialdehyde (MDA), a by-product of this process [11].

When oxidative stress occurs, plants employ various mechanisms to protect themselves and minimize damage. One such mechanism is the activation of ROS-scavenging antioxidant enzymes. Their activity is crucial not only during acute drought stress but also plays a key role in recovery [13]. Superoxide dismutase (SOD) is often regarded as a “first line of defense” against oxidative stress, as it scavenges hydrogen peroxide (H_2_O_2_) and superoxide ions (O2^•−^) to maintain redox balance [10,11]. In addition to SOD, several other antioxidant enzymes, such as catalase (CAT), peroxidase (POD), and ascorbate peroxidase (APX), also help defend against oxidative stress caused by drought. Catalase plays a crucial role in preventing cellular oxidative damage by efficiently breaking down H_2_O_2_ into water and oxygen [14]. Similarly, APX reduces H_2_O_2_ to water by using ascorbate (ASC) as an electron donor in various subcellular compartments [15]. Peroxidases (POD) are also commonly found in plants, where they help protect cells from oxidative stress and damage caused by H_2_O_2_ [16]. The activity of these antioxidant enzymes depends on the plant species, as well as the intensity and duration of the stress. Some antioxidants act as effective ROS scavengers during moderate stress, while their activity decreases under high stress intensity, and for others, the opposite is true. For example, the activation of CAT and APX is stronger in drought-tolerant species compared to sensitive ones [13]. A high antioxidant capacity to neutralize toxic ROS has been associated with increased plant tolerance to environmental stresses [17].

Previous studies have shown that fertilization can reduce the negative effects of drought on overall plant growth and development, partly by positively influencing antioxidant enzyme activities [11]. In our study, we focused on phosphorus (P) fertilization, a macronutrient that regulates various metabolic processes in plants, which in turn affects their physiological functions and growth [18]. Research on the impact of P fertilization on the antioxidant capacity of plants, particularly forest trees, is relatively limited. Phosphorus addition may help regulate photo-assimilates and maintain membrane integrity, thereby reducing the negative impact of drought stress [19]. Furthermore, Iqbal et al. [8] emphasized that P fertilizer could improve plant tolerance to stress through positive adjustments in morphological, physiological, and biochemical processes, while Ullah et al. [20] showed a positive effect of P through the reduction in MDA concentration, i.e., the rate of lipid peroxidation. Tariq et al. [19,21] studied the impact of P fertilization on the antioxidant system of three species of forest trees, concluding that P positively influences antioxidant capacity and reduces ROS production during drought stress.

Common beech (*Fagus sylvatica* L.) is the most widespread tree species in Central Europe, often growing alongside sessile oak (*Quercus petraea* (Matt.) Liebl.) [22]. Numerous studies have shown that beech is more sensitive to drought than oak [1,23,24]. Additionally, it has been established that different provenances of various species exhibit varying phenotypic plasticity in their response to drought [2,25]. Therefore, it is plausible that provenances also differ in their ability to mitigate oxidative stress caused by drought. Previous studies on sessile oak and pedunculate oak (*Quercus robur* L.) have shown that offspring from humid old-growth stands accumulate more antioxidants under favorable conditions but deplete them during drought, whereas saplings from arid old-growth stands maintain their antioxidant capacity even under harsher environmental conditions [26]. Similarly, in the Asian oak species *Quercus brantii* (Lindl.), drought stress increased the activity of antioxidant enzymes like POD, SOD, CAT, and APX more in plants from arid provenances compared to those from wetter ones, while the concentration of malondialdehyde (MDA) rose more in plants from wetter provenances [27]. In another Asian tree species, plants from arid provenances exhibited higher SOD and POD activity under moderate to severe drought stress compared to those from wetter regions, suggesting enhanced drought tolerance through increased antioxidant enzyme activity [28]. Our study was conducted on saplings from two provenances. To the best of our knowledge, no published research has examined whether phosphorus (P) fertilization can enhance drought tolerance in common beech and sessile oak by affecting antioxidant enzyme activity, or whether there are differences between provenances in this response. Therefore, the aims of this research were as follows:

1. To investigate the impact of drought, phosphorus fertilization, and provenance on the antioxidative capacity of common beech and sessile oak;
2. To assess the impact of phosphorus fertilization under drought conditions on the antioxidative capacity in common beech and sessile oak;
3. To investigate the differences in antioxidative capacity in common beech and sessile oak provenances.

## 2. Results

### 2.1. Water Availability in Soil and Leaf Water Potential

**Figure 1:**
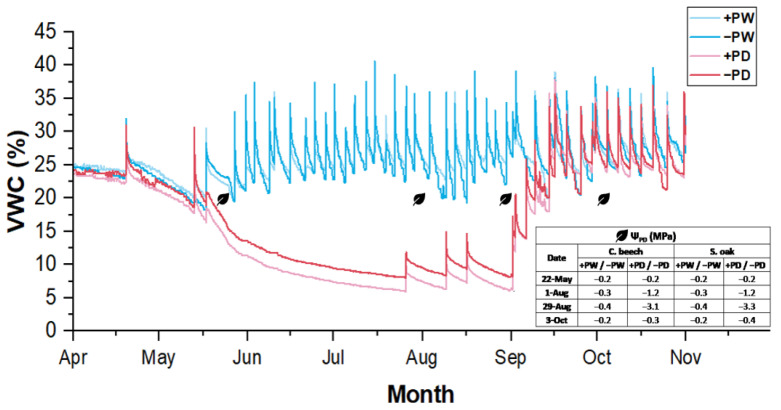
Patterns of substrate volumetric water content (VWC) in four treatments: regularly watered with phosphorus (+PW), regularly watered without phosphorus (−PW), drought with phosphorus (+PD), and drought without phosphorus (−PD), with mean pre-dawn leaf water potential (Ψ_PD_) in regularly watered (+PW and −PW) and drought-treated (+PD and −PD) common beech (C. beech) and sessile oak (S. oak) saplings, measured on 22 May, 1 and 29 August as well as on 3 October.

During the growing season, the volumetric water content (VWC) in the regularly watered treatments ranged from 18.3% to 40.6%. In contrast, the drought treatments experienced a continuous decline in VWC, reaching low levels of 8.0% and 10.1% by the end of August, marking the peak of the drought (Figure 1). Regular watering had a significant positive impact on the pre-dawn leaf water potential (Ψ_PD_) of both common beech and sessile oak. In the regularly watered treatments, Ψ_PD_ remained above −0.4 MPa throughout the season. However, in the drought treatments, Ψ_PD_ decreased steadily, hitting its lowest value on 29 August, with beech at −3.1 MPa and oak at −3.3 MPa (Figure 1).

### 2.2. Leaf Phosphorus Concentrations

**Figure 2:**
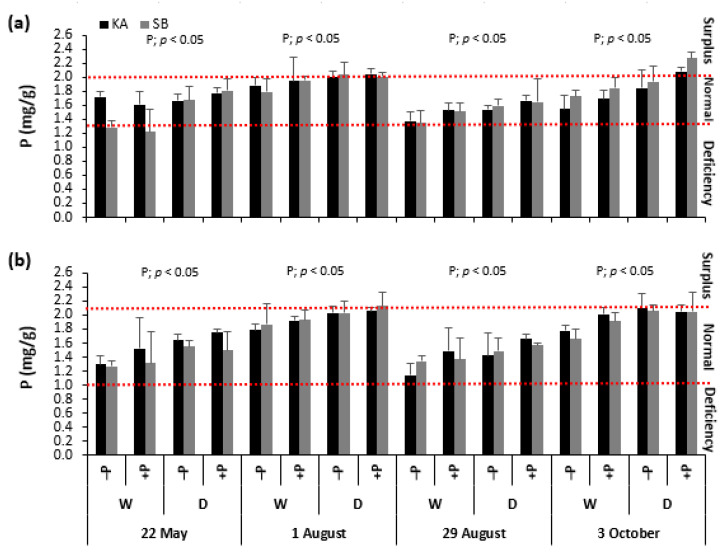
Mean values ±SE of phosphorus (P) concentrations in the leaves in response to drought (D) and regularly watered (W) treatments, as well as to phosphorus fertilization (+P) and non-fertilization (−P) treatments, for common beech (**a**) and sessile oak (**b**) from the Karlovac (KA) and Slavonski Brod (SB) provenances, measured on 22 May, 1 and 29 August, and 3 October. The figures show a significant effect of phosphorus fertilization (P) on P concentrations in the leaves, as determined by factorial ANOVA (*p* < 0.05). Red horizontal dashed lines represent the critical phosphorus concentrations in the leaves of common beech and sessile oak saplings [29].

Phosphorus fertilization significantly impacted leaf phosphorus concentrations (P_leaves_) in both beech and oak (Figure 2a,b). For beech fertilized with P, P_leaves_ ranged from 1.79 to 1.97 mg P g^−1^ DW, reflecting a nutritional status between upper normal and surplus (Figure 2a). In contrast, oak had P_leaves_ ranging from 1.92 to 1.99 mg P g^−1^ DW, indicating upper normal nutrition (Figure 2b). In non-fertilized beech, P_leaves_ ranged from 1.49 to 1.69 mg P g^−1^ DW, suggesting a mid-normal nutritional status. Meanwhile, non-fertilized oak had P_leaves_ between 1.41 and 1.55 mg P g^−1^ DW, indicating a lower normal nutrition level.

### 2.3. Photosynthetic Pigments

**Figure 3:**
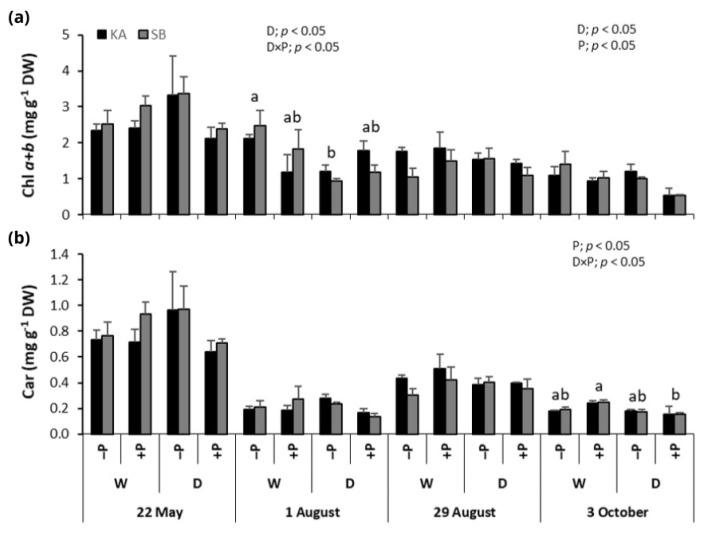
Mean values ±SE of (**a**) chlorophyll *a* + *b* (Chl *a* + *b*) and (**b**) carotenoid (Car) concentrations in response to drought (D) and regularly watered (W) treatments, as well as to phosphorus fertilization (+P) and non-fertilization (−P) treatments, for common beech from the Karlovac (KA) and Slavonski Brod (SB) provenances, measured on 22 May, 1 and 29 August, and 3 October. The figures show significant effects of drought (D), phosphorus fertilization (P), provenance (Pr), and the drought × phosphorus fertilization interaction (D × P) on Chl *a* + *b* and Car, as determined by factorial ANOVA (*p* < 0.05). Lowercase letters indicate significant differences among treatments.

Chlorophyll *a* + *b* (Chl *a* + *b*) concentrations in beech leaves peaked on 22 May and declined through the growing season (Figure 3a). Drought significantly reduced Chl *a* + *b* on 1 August and 3 October, with lower concentrations in drought (+PD and −PD) compared to regularly watered (+PW and −PW) treatments (Figure 3a). On 1 August, a drought ×P fertilization interaction showed significantly lower Chl *a* + *b* in the −PD treatment, which was not observed later in the season. On 3 October, the same interaction revealed lower Chl *a* + *b* in P-fertilized treatments (+PW and +PD) compared to non-fertilized treatments (−PW and −PD) (Figure 3a).

Carotenoid (Car) concentrations in beech leaves were highest on 22 May and decreased towards the season’s end. However, Car concentrations were higher on 29 August, during the peak of drought, compared to 1 August and 3 October (Figure 3b). Drought significantly affected Car concentrations at the end of the season, with regularly watered treatments (+PW and −PW) having higher Car than drought treatments (+PD and −PD) (Figure 3b). A significant drought × P fertilization interaction also showed higher Car concentrations in +PW compared to +PD treatments (Figure 3b).

**Figure 4:**
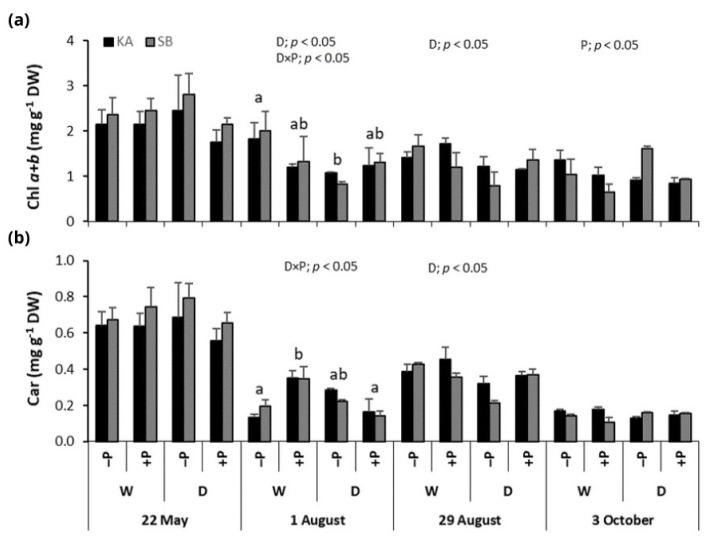
Mean values ±SE of (**a**) chlorophyll *a* + *b* (Chl *a* + *b*) and (**b**) carotenoid (Car) concentrations in response to drought (D) and regularly watered (W) treatments, as well as to phosphorus fertilization (+P) and non-fertilization (−P) treatments, for sessile oak from the Karlovac (KA) and Slavonski Brod (SB) provenances, measured on 22 May, 1 and 29 August, and 3 October. The figures show significant effects of drought (D), phosphorus fertilization (P), provenance (Pr), and the drought × phosphorus fertilization interaction (D × P) on Chl *a* + *b* and Car, as determined by factorial ANOVA (*p* < 0.05). Lowercase letters indicate significant differences among treatments.

The drought had a significant impact on Chl *a* + *b* concentrations in oak, particularly on 1 and 29 August when the regularly watered treatments (+PW and −PW) showed higher concentrations compared to the drought treatments (+PD and −PD) (Figure 4a). On 1 August, a significant drought × P fertilization interaction revealed that the −PW treatment had the highest Chl *a* + *b* concentrations compared to the other treatments. On 3 October, at the end of the growing season, oaks in the non-fertilized P treatments (−PW and −PD) had significantly higher Chl *a* + *b* concentrations than those in the P-fertilized treatments (+PW and +PD) (Figure 4a).

Similar to beech, Car concentrations in oak leaves were highest on 22 May and decreased toward the end of the season. Additionally, Car concentrations were higher on 29 August, during the peak of the drought, compared to both 1 August and 3 October (Figure 4b). On 1 August, a significant drought × P fertilization interaction showed the highest Car concentrations in the +PW treatment. Drought also significantly affected Car concentrations on 29 August, when the regularly watered treatments (+PW and −PW) had higher concentrations compared to the drought treatments (+PD and −PD) (Figure 4b).

### 2.4. Malondialdehyde Concentrations

**Figure 5:**
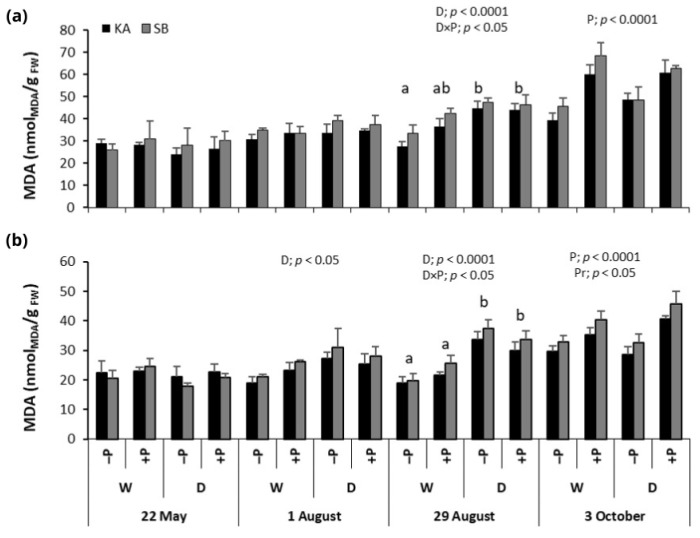
Mean values ±SE of malondialdehyde (MDA) concentrations in response to drought (D) and regularly watered (W) treatments, as well as to phosphorus fertilization (+P) and non-fertilization (−P) treatments, for common beech (**a**) and sessile oak (**b**) from the Karlovac (KA) and Slavonski Brod (SB) provenances, measured on 22 May, 1 and 29 August, and 3 October. The figures show significant effects of drought (D), phosphorus fertilization (P), provenance (Pr), and the drought × phosphorus fertilization interaction (D × P) on MDA, as determined by factorial ANOVA (*p* < 0.05). Lowercase letters indicate significant differences among treatments.

In beech, MDA concentrations generally increased from the beginning to the end of the growing season. At the peak of the drought period on 29 August, drought significantly increased MDA concentrations, with drought treatments (+PD and −PD) showing significantly higher levels compared to regularly watered (+PW and −PW) treatments (Figure 5a). Additionally, on 29 August, significant drought × P fertilization interaction revealed the lowest MDA concentration in −PW treatment compared to other treatments. By the end of the growing season, on 3 October, P fertilization led to higher MDA concentrations in P fertilized (+PW and +PD) compared to P non-fertilized treatments (−PW and −PD) (Figure 5a).

Malondialdehyde concentrations in oak followed the same pattern throughout the vegetation period as those in beech. However, drought significantly raised MDA concentrations on both 1 August and 29 August, with drought-exposed treatments (+PD and −PD) showing significantly higher MDA levels compared to regularly watered treatments (+PW and −PW) (Figure 5b). At the peak of the drought on 29 August, a significant interaction between drought and phosphorus fertilization was observed, with the −PW treatment exhibiting the lowest MDA concentrations. By the end of the growing season on 3 October, P fertilization led to higher MDA concentrations in P-fertilized treatments (+PW and +PD) compared to P non-fertilized treatments (−PW and −PD) (Figure 5b). Additionally, on 3 October, saplings from the SB provenance showed significantly higher MDA concentrations than those from the KA provenance.

### 2.5. Antioxidant Enzyme Activities in Common Beech

**Figure 6:**
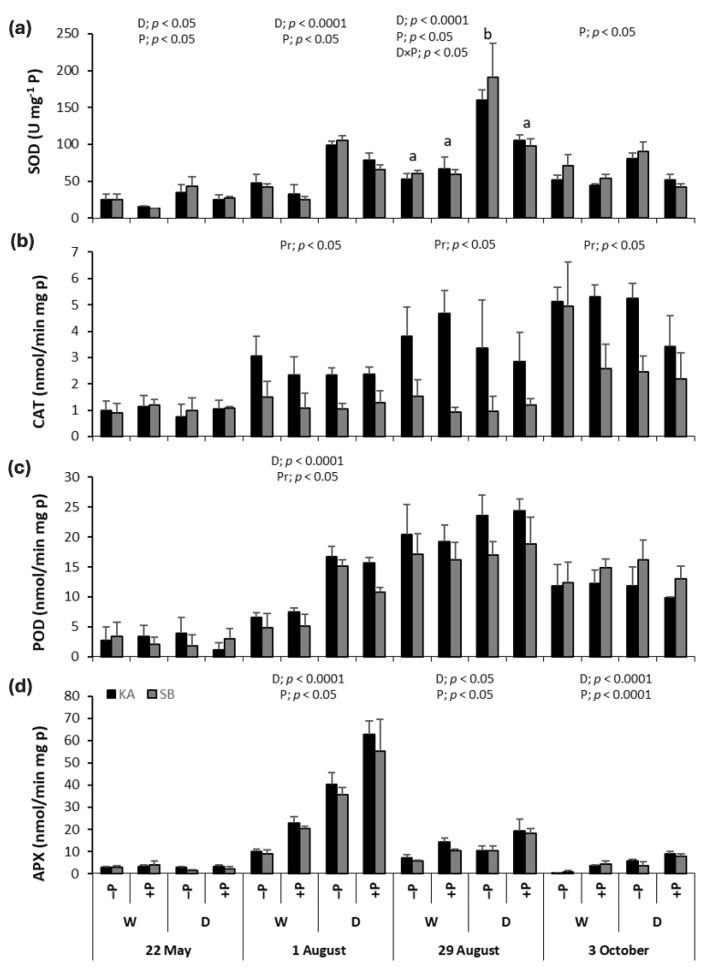
The mean values ± SE of (**a**) superoxide dismutase (SOD), (**b**) catalase (CAT), (**c**) peroxidase (POD), and (**d**) ascorbate peroxidase (APX) activity in response to drought (D) and regularly watered (W) treatments, as well as to phosphorus fertilization (+P) and non-fertilization (−P) treatments, for common beech from the Karlovac (KA) and Slavonski Brod (SB) provenances, measured on 22 May, 1 and 29 August, and 3 October. The figures show significant effects of drought (D), phosphorus fertilization (P), provenance (Pr), and the drought × phosphorus fertilization interaction (D × P) on SOD, CAT, POD, or APX activities, as determined by factorial ANOVA (*p* < 0.05). Lowercase letters indicate significant differences among treatments.

During the drought period, SOD activity was significantly higher in drought treatments (+PD and −PD) compared to regularly watered treatments (+PW and −PW). After the drought was released on 29 August, SOD activity decreased, with no significant difference between the drought and regularly watered treatments. Throughout the growing season, SOD activity was significantly higher in non-P-fertilized treatments (−PD and −PW) compared to P-fertilized treatments (+PD and +PW). A significant interaction between drought and P fertilization at the peak of the drought on 29 August revealed significantly higher SOD activity in the −PD treatment compared to the other treatments. SOD activity was similar across both provenances throughout the growing season (Figure 6a).

Drought and P fertilization did not significantly affect CAT activity during the growing season (Figure 6b). However, CAT activity was higher in the KA provenance compared to the SB provenance for almost the entire growing season, except at the very beginning (22 May). The highest CAT activities across all treatments and provenances were recorded on 3 October, at the end of the growing season (Figure 6b).

In all treatments and both provenances, POD activity increased until 29 August, the peak of the drought, after which it declined. On 1 August, POD activity was significantly higher in the drought treatments (+PD and −PD) compared to the regularly watered treatments (+PW and −PW) and in the KA provenance compared to the SB provenance (Figure 6c). Similar to CAT, P fertilization did not affect POD activity throughout the growing season.

APX activity increased sharply until 1 August, after which it declined sharply. Throughout almost the entire growing season, except at the very beginning (22 May), APX activity was significantly higher in drought treatments (+PD and −PD) compared to regularly watered treatments (+PW and −PW), and in P-fertilized treatments (+PW and +PD) compared to non-P-fertilized treatments (−PW and −PD). There were no significant differences between the provenances (Figure 6d).

### 2.6. Antioxidant Enzyme Activities in Sessile Oak

**Figure 7:**
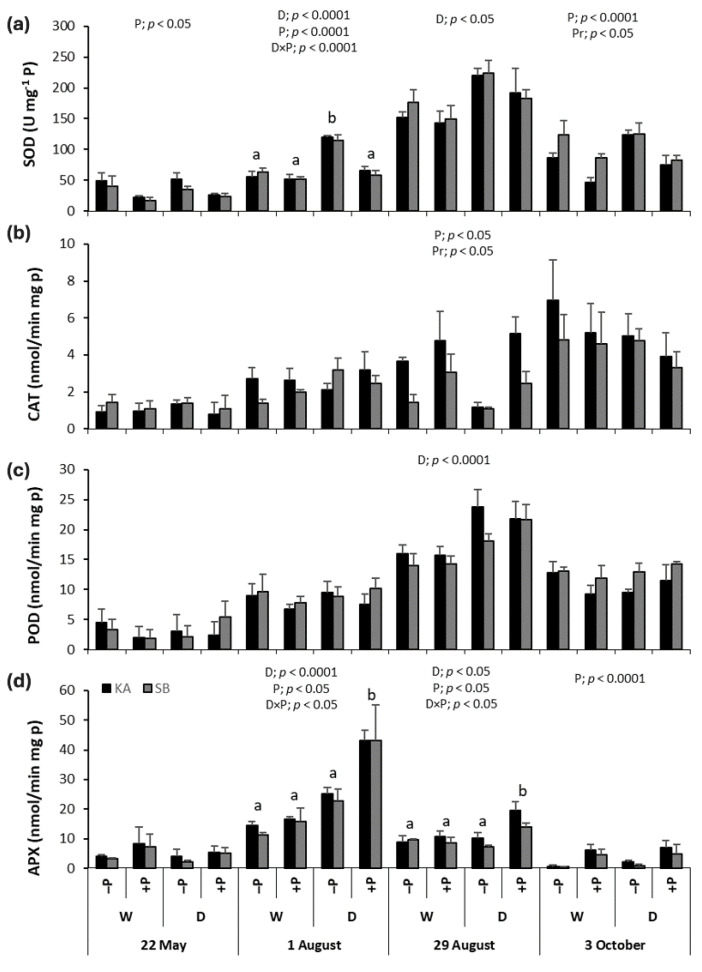
The mean values ± SE of (**a**) superoxide dismutase (SOD), (**b**) catalase (CAT), (**c**) peroxidase (POD), and (**d**) ascorbate peroxidase (APX) activity in response to drought (D) and regularly watered (W) treatments, as well as to phosphorus fertilization (+P) and non-fertilization (−P) treatments, for sessile oak from the Karlovac (KA) and Slavonski Brod (SB) provenances, measured on 22 May, 1 and 29 August, and 3 October. The figures show significant effects of drought (D), phosphorus fertilization (P), provenance (Pr), and the drought × phosphorus fertilization interaction (D × P) on SOD, CAT, POD, or APX activities, as determined by factorial ANOVA (*p* < 0.05). Lowercase letters indicate significant differences among treatments.

During the drought period (1 August and 29 August), SOD activity in the drought treatments (+PD and −PD) was significantly higher than in the regularly watered treatments (+PW and −PW) (Figure 7a). SOD activity was consistently lower in the P-fertilized treatments (+PD and +PW), compared to the non-P-fertilized treatments (−PW and −PD) throughout most of the season, except at the peak of the drought (29 August). On 1 August, a significant interaction between drought and P fertilization revealed significantly higher SOD activity in the −PD treatment compared to the other treatments. In all treatments, the highest SOD activity was recorded at the peak of the drought (29 August). Provenances showed significant differences only at the end of the season (3 October) when oaks from the KA provenance exhibited lower SOD activity compared to saplings from the SB provenance.

Generally, the highest CAT activity was recorded at the end of the growing season (3 October) (Figure 7b). On 29 August, at the peak of the drought, CAT activity was significantly higher in P-fertilized treatments (+PD and +PW) than in non-P-fertilized treatments (−PW and −PD) (Figure 7b). Provenances also differed significantly at this time, with saplings from the KA provenance showing higher CAT activity than those from the SB provenance.

POD activity was affected solely by drought, and only at the peak of the drought (29 August), when POD activity was significantly higher in the drought treatments (+PD and −PD) than in the regularly watered treatments (+PW and −PW) (Figure 7c). In all treatments, the highest POD activity occurred at the peak of the drought (29 August).

On 1 and 29 August, APX activity was higher in the drought treatments (+PD and −PD) compared to the regularly watered treatments (+PW and −PW) (Figure 7d). Throughout August and at the end of the season in October, APX activity was higher in the P-fertilized treatments (+PD and +PW) compared to non-P-fertilized treatments (−PW and −PD). A significant interaction between drought and P fertilization on 1 August and 29 August revealed significantly higher APX activity in the +PD treatment compared to the other treatments.

## 3. Discussion

### 3.1. Effect of Drought on Malondialdehyde Concentrations, Photosynthetic Pigments and Antioxidant Enzyme Activities

In both species, plants subjected to drought exhibited significantly higher malondialdehyde (MDA) concentrations at the peak of drought compared to regularly watered ones. This response is typical for plants under drought stress, indicating elevated rates of lipid peroxidation, as previously documented in both crops and forest trees [17,21,30,31]. These findings suggest that drought stress leads to increased oxidative stress and hydrogen peroxide (H_2_O_2_) accumulation, resulting in damage to cell membranes—often the primary targets of various biotic and abiotic stresses [12]. Consequently, this damage compromises plasma membrane integrity, causing significant harm to the cell membranes [10,11], thereby exacerbating the negative impacts of drought on the plants.

This oxidative damage is reflected in the significant reduction in photosynthetic pigment concentrations observed under drought stress. Chlorophyll *a* + *b* concentrations were notably lower in drought-exposed plants compared to regularly watered ones, although this was somewhat more pronounced in oak. Additionally, in beech, carotenoid concentrations were higher in regularly watered plants than in drought-exposed ones, a trend also observed in oak during the peak drought period in August. The reduction in photosynthetic pigments, including chlorophylls and carotenoids, impairs photosynthesis and severely limits primary production, as documented in several studies [5,10,19,20,21,32]. Such declines are typically linked to pigment photo-oxidation and chlorophyll degradation, with increasing reactive oxygen species (ROS) production during drought exacerbating these processes and impairing chlorophyll biosynthesis [5,33].

During drought conditions, the increased production of reactive oxygen species (ROS) leads to oxidative stress, making it essential for plants to have effective antioxidant defense mechanisms to mitigate the negative effects of drought. In this study, we focused on antioxidant enzymes responsible for scavenging excess ROS. Superoxide dismutase (SOD) activity significantly increased in both beech and oak under drought stress compared to regularly watered plants, a result consistent with previous studies on various crop and tree species [19,20,21,30,32,33,34]. While Chatterjee et al. [33] showed that SOD activity increases with light intensity but not with increasing drought in rice, our results indicate that both beech and oak exhibited the highest SOD activity precisely during peak drought stress. This suggests effective regulation of the oxidative stress defense mechanism, as SOD activity was maintained even under severe drought conditions.

Interestingly, catalase (CAT) activity did not show a significant change in response to drought in either species, which contrasts with findings from other studies where CAT activity was found to increase under drought stress in various crops and trees [10,20,21,30]. However, research on two oak species found that CAT activity significantly decreased during drought stress [35], while moderate water stress induced CAT activity in rice, and higher levels of water stress led to a decrease in activity [33]. Furthermore, in *Arabidopsis thaliana*, some mutants exhibited reduced CAT activity due to excessive lipid peroxidation and peroxisome destruction during drought stress [34]. In our study, the significant increase in MDA levels in drought-exposed plants suggests that severe lipid peroxidation may have caused too much damage, thereby limiting CAT’s role in mitigating oxidative stress.

Peroxidase (POD) activity, however, did respond to drought stress in both species. Higher POD levels were observed in drought-treated saplings compared to regularly watered ones in beech at the beginning of August and in oak at the peak of drought at the end of August. Similar findings have been reported in other studies [19,20,30,32], underscoring the important role of POD as an antioxidant defense mechanism in both beech and oak. However, in beech, no significant difference was found between drought-treated and regularly watered plants during the peak of drought stress, suggesting that POD’s effectiveness diminished under extreme drought conditions. Similar results have been reported in peanuts [36]. These results indicate that some antioxidant enzymes, like POD, may be more effective under moderate drought conditions but less so under severe stress, highlighting the variability of antioxidant responses depending on plant species and stress intensity.

As for ascorbate peroxidase (APX), we found that drought treatment throughout most of the growing season significantly increased APX activity in beech compared to regularly watered plants. In oak, higher APX activity was observed during moderate and peak drought periods in August. These results are consistent with findings from Chatterjee et al. in rice [33]. The highest APX activity occurred in early August, during moderate drought, when APX was most effective in defending against oxidative damage. However, by the peak of drought stress, APX activity decreased significantly in both species, suggesting that APX becomes less effective as an antioxidant under extreme drought conditions. This is in contrast to findings in peanuts, where APX activity remained high even under extreme drought stress [36], but mirrors the patterns observed for CAT and aligns with our hypothesis that severe lipid peroxidation during peak drought may inhibit APX’s protective role.

### 3.2. Effect of Phosphorus Fertilization and Drought × Phosphorus Fertilization Interaction on Malondialdehyde Concentrations, Photosynthetic Pigments and Antioxidant Enzyme Activities

It is hypothesized that fertilization with nitrogen (N) and phosphorus (P) could help plants cope with drought stress by regulating membrane integrity [37], thereby reducing lipid peroxidation rates and MDA concentrations. Previous research has shown that P fertilization positively affects MDA levels during drought, with lower MDA rates observed in P-fertilized plants compared to non-fertilized ones [19,21,30]. However, in our study, P fertilization did not significantly reduce MDA levels in either species. While oak plants from the −PD treatment had higher MDA concentrations at the peak of drought than those from the +PD treatment, the difference was not statistically significant. Additionally, at the end of the season, both species displayed significantly higher MDA concentrations in P-fertilized plants compared to non-fertilized ones. This was observed in early October, and we conclude that P fertilization did not benefit the plants; rather, it exacerbated membrane damage and accelerated leaf senescence as the growing season ended. This is also evident from the lower chlorophyll concentrations in both species in October in the P-fertilized treatments.

Our study could not conclude that P fertilization improved chlorophyll *a* + *b* concentrations in either beech or oak. A study on the tree species *Alnus cremastogyne* found no significant differences in photosynthetic pigment concentrations due to P application [19]. However, in *Eucalyptus grandis*, P application significantly increased chlorophyll concentrations in drought-exposed plants [21]. In our study, plants fertilized with P (+PD treatment) exhibited higher chlorophyll *a* + *b* levels compared to non-fertilized plants (−PD treatment) during periods of advanced drought (1 August, as shown in Figure 3a and Figure 4a), but this difference was not statistically significant. By the end of the season, chlorophyll *a* + *b* concentrations were lower in the P-fertilized plants than in the non-fertilized ones. It is possible that the severe drought conditions in our study masked any potential positive effects of P on drought tolerance in these species regarding photosynthetic pigment concentrations.

Interestingly, P fertilization did not enhance SOD activity in either species; in fact, it had the opposite effect. In August, the −PD treatment showed the highest SOD activity (at the beginning of August for oak and at the end for beech), while the +PD treatment did not differ from the regularly watered treatments. Thus, P fertilization seemed to decrease SOD activity, negatively impacting the antioxidant defense during drought. This finding contrasts with previous studies that reported positive effects of P on antioxidant metabolism in various tree species [19,21]. We believe our results arise from the fact that, although our saplings were not in P surplus, they were near that threshold (Figure 2). Takagi et al. [38] noted that high inorganic P accumulation can cause an over-reduction in the photosynthetic electron transport chain, leading to additional ROS production already excessive due to drought. Under such conditions, homeostasis may be disrupted, and the antioxidant system becomes ineffective, which is reflected in the reduced SOD activity observed in our research.

Fertilization did not affect CAT activity in beech, but in oak, P-fertilized plants in both drought and regularly watered treatments showed significantly higher CAT activity than non-fertilized ones during the drought peak. Previous studies have reported mixed results regarding P fertilization: in some cases, it decreased CAT activity in drought-stressed plants compared to non-fertilized ones [20,21], while in others, it increased CAT activity [19]. In our case, P fertilization was beneficial for drought-stressed oak, as it stimulated greater CAT activity, enhancing the plants’ defense against oxidative damage from drought. As for POD, P fertilization did not affect its activity in either species. The results of previous studies are quite varied: some studies found no effect of P on POD activity during drought, as in our case [30], while others reported increased POD activity in drought-stressed plants due to P fertilization [19], and some found that P reduced POD activity during drought [21]. Therefore, based on our findings and previous studies, we cannot conclude whether P fertilization improves plant responses to oxidative stress in terms of POD activity.

Phosphorus fertilization did, however, significantly enhance APX activity in both species throughout most of the growing season, except for the early stages, as evidenced by the higher APX activity in plants from fertilized treatments compared to those from non-fertilized treatments. In oak, the positive effect of P on APX activity during drought stress was most pronounced in August, when the +PD treatment showed the highest APX activity, significantly differing from all other treatments. Laxa et al. highlighted that APX activation is stronger in drought-tolerant species compared to less tolerant ones [13], a finding that aligns with our results, as oak is generally regarded as a drought-tolerant species [6].

### 3.3. Effect of Provenance on the Malondialdehyde Concentrations, Photosynthetic Pigments, and Antioxidant Enzyme Activities

Previous research has shown that provenances vary in response to drought in the context of adaptive antioxidant defense mechanisms. For example, in one oak species, MDA levels increased more in plants from wetter provenances than those from drier provenances [27]. In our study, oaks from the KA provenance exhibited significantly lower MDA levels than those from the SB provenance at the end of the growing season. This may indicate better adaptation or recovery from drought stress, consistent with our previous findings that oak from the KA provenance demonstrated better photosynthetic performance, as indicated by higher net photosynthesis rates (A_net_) and photosynthetic performance index (PI_abs_) compared to those from the SB provenance [39]. We believe this is due to the drier growing conditions in the natural habitats of KA provenance (2016–2020), which may have enhanced their resilience to drought, showcasing a significant degree of intra-specific diversity. Additionally, previous studies on two tree species have shown that plants from drier provenances had higher antioxidant enzyme activity compared to plants from wetter provenances [27,28]. In our research, beech from the KA provenance exhibited higher CAT activity compared to the SB provenance throughout most of the season; a similar trend was observed in oak during the peak of the drought. This suggests that KA provenance plants are better adapted to oxidative stress caused by drought due to their enhanced CAT activity. Beech from the KA provenance also exhibited higher POD activity compared to the SB provenance during the advanced drought period (early August). We believe this is due to the same factors observed in previous cases, indicating that saplings from KA are better adapted to drought stress in various ways than those from SB. These results suggest that in our study, provenances that experienced lower precipitation during the growth period of the investigated saplings showed better drought resistance, reflected by higher antioxidant enzyme activity and lower MDA concentrations compared to provenances with higher precipitation.

## 4. Materials and Methods

### 4.1. Plant Material and Provenance Habitat Conditions

**Figure 8:**
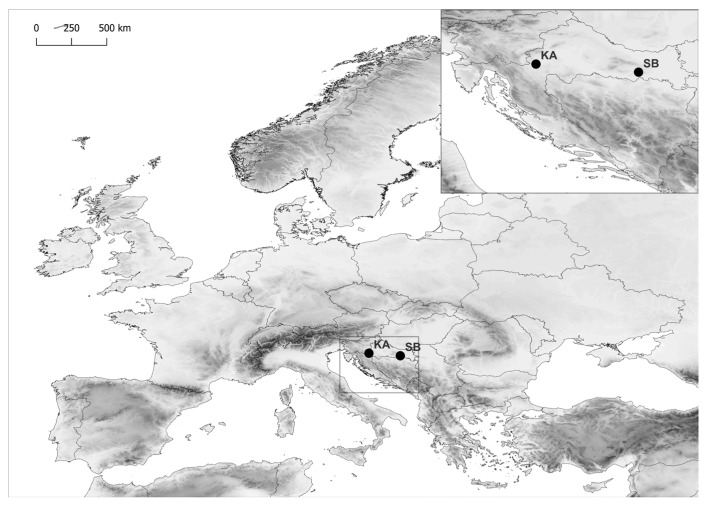
The geographic locations of the Slavonski Brod (SB) and Karlovac (KA) provenances.

This study focused on four-year-old saplings of common beech (*Fagus sylvatica* L.) and sessile oak (*Quercus petraea* (Matt.) Liebl.) from two mature mixed stands in the continental region of the Republic of Croatia. One provenance was located in the north-western part of Croatia near Karlovac (KA provenance, 15.524041° E, 45.466135° N 170 m a.s.l.), with an approximate age of 100 years, while the second provenance was near Slavonski Brod in the eastern part of the country (SB provenance, 17.973173° E, 45.273451° N, 245 m a.s.l.), with an approximate age of 105 years (Figure 8). Both sites were dominated by these species.

In early March 2021, four-year-old saplings were carefully excavated from beneath mature trees, ensuring minimal disturbance to their root systems. The trees were spaced at least 100 m apart. Further details on the habitat characteristics of the studied provenances, including phytosociological, geomorphological, climatological, meteorological, and soil properties, can be found in [40]. Notably, the mean annual precipitation from 1949 to 2019 was higher in the KA (1111.8 mm) compared to the SB provenance (770.3 mm). However, between 2016 and 2020, during the growth period of the saplings, the KA provenance experienced 17 months of moderately to extremely dry months (9 of which occurred during the growing season), while the SB provenance experienced only 9 such months (4 during the growing season). During the experiment, the average daily temperature was 18.8 °C during the vegetation season (April–October), while the average daily relative humidity was 66.7%, and the average daily insolation was 7.6 h. Soil characteristics were largely similar between the two provenances. Phosphorus soil concentrations were low in both provenances with the KA provenance having a P_2_O_5_ concentration of 0.50 ± 0.32 mg/100 g of soil and the SB provenance having 0.64 ± 0.21 mg/100 g of soil, at a depth of 0–30 cm.

### 4.2. Experimental Design and Growth Conditions

The four-year-old saplings, averaging 36.6 ± 8.01 cm in height, were relocated to the garden of the Faculty of Forestry and Wood Technology at the University of Zagreb (45.82065 N, 16.02303 E, 120 m a.s.l.), where a common garden experiment was set up. The saplings were transplanted into four wooden boxes (155 × 275 × 80 cm, with a total volume of 3.41 m^3^) filled with 3800 L of Klasmann TS 3 substrate, which had a P_2_O_5_ concentration of 160 mg/L. To raise the phosphorus concentration to 300 mg/L, two of the four boxes received 1182 g of triple superphosphate (Triplex), a fertilizer containing 45% P_2_O_5_, while the other two boxes remained unfertilized, maintaining a moderate P_2_O_5_ level of 160 mg/L.

Each box was planted with 100 saplings, consisting of 50 common beech and 50 sessile oak saplings, or 25 saplings per species from each provenance (KA and SB), for a total of 400 saplings in the experiment. The saplings were arranged randomly with a spacing of 20 × 18 cm. During the 2021 growing season, the saplings were exposed to natural weather conditions and were regularly watered in the summer to aid acclimatization and survival. In 2022, a transparent PVC roof covered all boxes to prevent natural precipitation, and four treatments were applied. Two boxes, one fertilized and one unfertilized with P, were watered manually with 40 L of water every four days. The other two boxes, also one fertilized and one unfertilized with P, were subjected to drought from 15 May to 1 September 2022. During this period, they were watered minimally (20 L per box) only when wilting leaves indicated drought stress, occurring three times: late July, early August, and mid-August.

The four experimental treatments were as follows: regular watering with P fertilization (+PW), regular watering without P fertilization (−PW), drought with P fertilization (+PD), and drought without P fertilization (−PD).

### 4.3. Soil Volumetric Water Content and Leaf Water Potential

The seasonal water dynamics in the substrates of all treatments were monitored using a data logger and sensors to measure the volumetric water content (VWC) in the soil (Spectrum Technologies, Inc., Aurora, CO, USA). Four sensors were installed at a depth of 5–20 cm in each treatment. Pre-dawn leaf water potential (Ψ_PD_) was measured on three randomly selected saplings of common beech and sessile oak per provenance in each treatment (12 saplings per treatment) using a portable pressure chamber (Model 600, PMS Instrument Company, Albany, OR, USA). The first measurements were taken on May 26 (onset of the drought period), followed by early August (moderate drought), late August (drought peak), and October (at the end of the growing season).

### 4.4. Leaf Sampling for Chemical Analysis

During the growing season, we collected three composite samples per species and provenance within each treatment. For phosphorus concentrations, concentrations of photosynthetic pigments, total soluble proteins, malondialdehyde, and antioxidant enzymes, we sampled in May, early August, late August, and October. On each occasion, the composite sample consisted of 10 leaves, which were then analyzed. In total, there were three composite samples for beech SB, three for beech KA, three for oak KA, and three for oak SB in each treatment, for a total of forty-eight samples per date.

### 4.5. Leaf Phosphorus Concentration

Three composite leaf samples were collected in May, early August, late August and October to analyze phosphorus concentration. The leaves were then dried, ground, and homogenized before undergoing chemical analysis. The phosphorus concentration was determined using a spectrophotometric method, following standardized international protocols [41].

### 4.6. Chlorophyll and Carotenoid Concentrations

The leaves were ground and homogenized in a mortar with liquid nitrogen. From 0.1 g of the prepared powder, Eppendorf cuvettes of 0.2 mL volume were filled with the addition of cold acetone (80%). The tissue was then extracted for 15 min at 4 °C, after which it was centrifuged at 1000 rpm for 10 min. The supernatant was decanted into plastic test tubes, while the green-colored solid residue was re-extracted with acetone until the tissue lost its green color. The volume of each sample was measured using a graduated cylinder.

The prepared samples were used for spectrophotometric measurements on a UV/VIS spectrophotometer (Specord 40, Analytic Jena, Jena, Germany). Absorbance was measured at wavelengths of 661.6 nm, 664.8 nm, and 470 nm. Based on the known leaf mass (m), volume (V), and absorbance (A) at each wavelength, the concentrations of photosynthetic pigments (chlorophyll *a*, chlorophyll *b*, chlorophyll *a* + *b*, and carotenoids) were calculated using the following formulas according to [42]:

Chlorophyll *a* = (11.24 × A (661.6 nm) − 2.04 × A (664.8 nm)) × V/(m × 1000),

Chlorophyll *b* = (20.13 × A (664.8 nm) − 4.19 × A (661.6 nm)) × V/(m × 1000),

Chlorophyll *a* + *b* = (7.05 × A (661.6 nm) + 18.09 × A (664.8 nm)) × V/(m × 1000),

Carotenoids = (1000 × A (470 nm) − 1.90 × (11.24 × A (661.6 nm) − 2.04 × A (664.8 nm)) − 63.14 × (20.13 × A (664.8 nm) − 4.19 × A (661.6 nm))) × V/(214 × m × 1000)

The measured concentrations of photosynthetic pigments were expressed in mg/g fresh weight (FW) of the sample.

### 4.7. Determination of Malondialdehyde Concentration

To determine the concentration of lipid peroxidation, leaf samples were extracted and homogenized in a mortar. A solution of trichloroacetic acid (TCA) in water was added to the homogenate, after which the sample was centrifuged (100,000 rpm, 5 min). The supernatant was used to measure lipid peroxidation concentration, based on the quantification of malondialdehyde (MDA), the final product of lipid peroxidation. To 1 mL of supernatant, 1 mL of 0.5% thiobarbituric acid (TBA) in 20% TCA was added. The mixture was heated for 30 min in a water bath at 90 °C, then rapidly cooled and centrifuged (10,000 rpm, 10 min). Absorbance was measured at 390 nm, and MDA was expressed as nmol per gram of fresh tissue (nmol g^−1^ fresh weight).

### 4.8. Determination of Protein Concentration and Antioxidant Enzyme Activity

Plant leaves were homogenized on ice using a mortar and pestle. For extraction, plant material was first weighed, then homogenized with the addition of 2 mL of extraction buffer (pH = 7.0) and polyvinylpolypyrrolidone (PVP, a protein stabilizer). After homogenization, the extract was centrifuged at 10,000 rpm, decanted, and stored at −20 °C until further analysis. The obtained extract was used for determining the total soluble protein concentration by the Bradford method and for measuring the activity of ascorbate peroxidase (APX), catalase (CAT), peroxidases (POD), and superoxide dismutase (SOD).

#### 4.8.1. Determination of Total Soluble Protein Concentration by Bradford Method

The concentration of total soluble proteins was determined using the Bradford method [43]. A 96-well plate was prepared by adding 10 μL of the sample to each well, followed by the addition of 225 μL of Bradford reagent. The plate was incubated for 15 min in the dark, and absorbance was measured spectrophotometrically at 595 nm.

#### 4.8.2. Measurement of Ascorbate Peroxidase Activity

The activity of ascorbate peroxidase (APX) was measured according to the protocol of Ambriović Ristov et al. [44]. In a quartz cuvette, 970 μL of APOX assay buffer was added, followed by 10 μL of sodium ascorbate, 10 μL of the extract, and 10 μL of hydrogen peroxide, initiating the reaction. The reaction progress was monitored spectrophotometrically at 290 nm every 15 s for 1 min. Ascorbate peroxidase activity was determined by the reduction in ascorbate, with an extinction coefficient of 2.8 mM^−1^ cm^−1^, and was expressed as nmol per minute per mg of total protein (nmol min^−1^ mg^−1^ P).

#### 4.8.3. Measurement of Catalase Activity

Catalase (CAT) activity was measured spectrophotometrically at 290 nm every 5 s for 1 min. The measurement was initiated by adding 5 μL of the diluted sample to 995 μL of catalase assay buffer [44]. The extinction coefficient is 40 mM^−1^ cm^−1^, and catalase activity was expressed as nmol per minute per mg of total protein (nmol min^−1^ mg^−1^ P).

#### 4.8.4. Measurement of Peroxidase Activity

Peroxidase (POD) activity was measured in a 96-well plate by adding 190 μL of POD assay buffer to each well, followed by the addition of 10 μL of the extract. Absorbance was measured every 15 s for 5 min at 470 nm. During the reaction, tetraguaiacol was formed, which has a molar extinction coefficient of 26.6 mM^−1^ cm^−1^. POD activity was expressed as mmol per minute per mg of total protein (nmol min^−1^ mg^−1^ P).

#### 4.8.5. Measurement of Superoxide Dismutase Activity

For the measurement of superoxide dismutase (SOD) activity, various dilutions of the extract were prepared by mixing specific volumes of the extract and protein extraction buffer (pH = 7.0) to a final volume of 33.3 μL in 96-well plates. To each well, 300 μL of SOD assay buffer (pH = 7.8) and 0.67 μL of 2 μM riboflavin were added, and the reaction was initiated by exposing the samples to light from a 36 W light source. The reaction lasted for 10 min, after which it was stopped by darkening the samples. SOD activity was measured spectrophotometrically at 560 nm 5–10 min after darkening. Enzyme activity was expressed as substrate reduction inhibition. Inhibition was calculated using the formula:

NBT (%) = [(A − B)/A] · 100,

where A is the absorbance measured after the reaction without enzyme (maximum absorbance value) and B is the absorbance measured after the reaction with enzyme (reduced absorbance). The enzyme activity curve was constructed by plotting the NBT reduction inhibition (%) on the *y*-axis and the volume of the extract used to prepare the dilutions on the *x*-axis. Based on this curve, the volume of extract required for 50% NBT inhibition in the reaction mixture was used to calculate the specific SOD activity, which was expressed in enzyme activity units per milligram of total protein (U mg^−1^ P).

### 4.9. Statistical Analysis

All statistical analyses were performed using SAS 15.1 software (SAS Institute Inc., Cary, NC, USA). The assumptions of residual normality and homogeneity of variances were assessed using the Shapiro-Wilk test and Levene’s test, conducted through the GLM and UNIVARIATE procedures in SAS. Residuals were plotted against fitted values to check for homogeneity of variance, and the residual distribution was also examined. A factorial ANOVA was used to assess the fixed effects of drought, phosphorus fertilization, provenance, and their interactions on leaf water potential, phosphorus concentrations in leaves, total chlorophyll and carotenoid concentrations, protein concentrations, malondialdehyde concentrations, and antioxidant enzyme activities (APX, CAT, POD, SOD) for each species individually. In all cases, a Tukey post hoc test was performed to determine the significance of differences (*p* < 0.05) between the levels of the factors studied.

## 5. Conclusions

Our study highlights the complex interactions between drought stress, phosphorus fertilization, and provenance in shaping the antioxidative capacity of common beech and sessile oak. Both species exhibited increased malondialdehyde (MDA) concentrations, a marker of lipid peroxidation and membrane damage, along with reduced photosynthetic pigments under drought conditions. While antioxidant enzyme activity increased under drought conditions, the effectiveness of these enzymes varied depending on the species, provenance, and the severity of the drought stress. Phosphorus fertilization did not consistently enhance drought tolerance, but it did affect antioxidant activity, particularly in oak, where increased catalase (CAT) and ascorbate peroxidase (APX) activities were observed during the peak of the drought. The results also revealed notable differences between provenances, with plants from the provenance that experienced drier conditions during the growth of the studied saplings in natural stands showing better adaptation to drought stress, as reflected in higher antioxidant enzyme activity and lower oxidative damage. Overall, the findings suggest that phosphorus fertilization may not always alleviate the negative effects of drought and that provenance-specific differences in antioxidant capacity can influence species’ resilience to drought stress. These findings underscore the complexity of plant responses to environmental stress and the potential role of provenance in shaping drought tolerance.

## Data Availability

The data in this study are available from the corresponding author upon request.
